# Gender Disparity in National Institutes of Health Funding Among Surgeon-Scientists From 1995 to 2020

**DOI:** 10.1001/jamanetworkopen.2023.3630

**Published:** 2023-03-20

**Authors:** Mytien Nguyen, Luis Gonzalez, Sarwat I. Chaudhry, Nita Ahuja, Bohdan Pomahac, Ashley Newman, Ashley Cannon, Shenika A. Zarebski, Alan Dardik, Dowin Boatright

**Affiliations:** 1 Yale School of Medicine, New Haven, Connecticut; 2 Section of General Internal Medicine, Department of Medicine, Yale School of Medicine, New Haven, Connecticut; 3 Department of Surgery, Yale School of Medicine, New Haven, Connecticut; 4 Department of Pathology, Yale School of Medicine, New Haven, Connecticut; 5 Department of Biological and Biomedical Sciences, Yale School of Medicine, New Haven, Connecticut; 6 Division of Plastic and Reconstructive Surgery, Yale School of Medicine, New Haven, Connecticut; 7 Howard University School of Medicine, Washington, DC; 8 Technical Resources International, Bethesda, Maryland; 9 Ohio University Heritage College of Osteopathic Medicine, Dublin; 10 Department of Surgery, VA Connecticut Healthcare System, West Haven; 11 Department of Emergency Medicine, New York University Grossman School of Medicine, New York, New York

## Abstract

**Importance:**

Surgical diseases account for approximately 30% of the global burden of disease. Gender diversity in biomedical research is critical to generate innovative patient-centered research in surgery.

**Objective:**

To examine the distribution of biomedical research funding by the National Institutes of Health (NIH) among women and men surgeon-scientists during a 25-year period.

**Design, Setting, and Participants:**

This cross-sectional study used publicly available data from the NIH RePORTER (Research Portfolio Online Reporting Tools: Expenditures and Results) database for research project grants awarded to women and men surgeon-scientists who were principal investigators between 1995 and 2020. Data were retrieved between January 20 and March 20, 2022. The representation of women surgeon-scientists among academic surgeons was compared with the representation of men surgeon-scientists over time.

**Main Outcomes and Measures:**

Distribution of NIH funding to women and men surgeon-scientists was examined via 2 metrics: holding a large-dollar (ie, R01-equivalent) grant and being a super principal investigator (SPI) with $750 000 or more in total annual research funding. Statistical analysis was performed between April 1 and August 31, 2022.

**Results:**

Between 1995 and 2020, 2078 principal investigator surgeons received funding from the NIH. The proportion of women academic surgeons who were surgeon-scientists remained unchanged during this same period (1995, 14 of 792 [1.8%] vs 2020, 92 of 3834 [2.4%]; *P* = .10). Compared with their men counterparts, women surgeon-scientists obtained their first NIH grant earlier in their career (mean [SD] years after first faculty appointment, 8.8 [6.2] vs 10.8 [7.9] years; *P* < .001) and were as likely to obtain large-dollar grants (aRR, 0.99 [95% CI, 0.95-1.03]) during the period 2016 to 2020. Despite this success, women surgeon-scientists remained significantly underrepresented among SPIs and were 25% less likely to be an SPI (aRR, 0.75 [95% CI, 0.60-0.95] during the period 2016 to 2020).

**Conclusions and Relevance:**

The findings of this cross-sectional study of NIH-funded surgeons suggest that women surgeons remained underrepresented among surgeon-scientists over a 25-year period despite early career success in receiving NIH funding. This is concerning and warrants further investigation to increase the distribution of NIH funding among women surgeon-scientists.

## Key Points

**Question:**

What was the gender distribution of National Institutes of Health funding among surgeon-scientists between 1995 and 2020?

**Findings:**

This cross-sectional study of 2078 surgeon-scientists who received National Institutes of Health funding between 1995 and 2020 found that despite an increase in proportion of women surgeon-scientists, women surgeons remained significantly underrepresented. Compared with surgeon-scientists who are men, women surgeon-scientists were significantly less likely to hold $750 000 or more in annual research funding.

**Meaning:**

These findings suggest that substantial additional support for women surgeon-scientists is necessary to achieve a gender-diverse surgical research workforce.

## Teaser

This cross-sectional study compares the distribution of National Institutes of Health (NIH) research project grants among surgeon-scientists over a 25-year period by gender.

## Introduction

Surgeon-scientists are critically centered at the intersection between patient care and biomedical sciences to drive innovation in health care. Prior studies have shown that women scientists are more likely to produce innovative work^1^; therefore, gender equity among surgeon-scientists may be imperative for clinical innovation. However, while the number of women medical students and surgery residents has increased in the past decade,^2^ trends in the gender composition of the surgeon-scientist workforce remain poorly understood.

The National Institutes of Health (NIH) is the largest funder of biomedical research in the US, and NIH funding confers many career advantages and prestige for surgeons. NIH-funded surgeons are more likely to be promoted to a higher academic rank, more likely to be elected to leadership positions in specialty societies, and more academically productive than surgeons without NIH funding.^3,4^ Furthermore, among NIH-funded investigators, those who are well funded may hold considerable career security and influence on policy making at both institutional and national levels.^5^ Therefore, obtaining NIH funding, especially large-dollar grants, confers prestige on academic surgeons. However, women surgeons have been historically underrepresented among NIH-funded surgeons^6^ and are less likely to benefit from these advantages.

Understanding temporal trends in NIH funding among women surgeon-scientists is critical to achieving gender parity among all surgeon-scientists to promote clinical innovation. In this study, we explored the gender diversity of NIH-funded academic surgeons from 1995 to 2020 and compared grant dollar funding success by gender. We hypothesized that over the 25-year study period, women surgeon-scientists would remain underrepresented among surgeon-scientists and would lag behind men surgeon-scientists in receiving large-dollar grants.

## Methods

This cross-sectional study was deemed exempt from review and informed consent by the Yale University Institutional Review Board owing to the use of publicly available data. The study followed the Strengthening the Reporting of Observational Studies in Epidemiology (STROBE) reporting guideline.

### Data Source

Data on NIH-funded principal investigators were obtained from the NIH RePORTER (Research Portfolio Online Reporting Tools: Expenditures and Results) database^7^ for the period 1995 to 2020; these data were retrieved between January 20 and March 20, 2022. Surgical department affiliations of principal investigators were included in the initial search and included the following specialties: surgery, orthopedic surgery, otolaryngology, plastic surgery, ophthalmology, neurosurgery, and urology. Research project grants included grants with the following activity codes, which include both small and large research project grants: DP1, DP2, DP3, DP4, DP5, P01, PN1, PM1, R00, R01, R03, R15, R21, R22, R23, R29, R33, R34, R35, R36, R37, R61, R50, R55, R56, RC1, RC2, RC3, RC4, RF1, RL1, RL2, RL9, RM1, UA5, UC1, UC2, UC3, UC4, UC7, UF1, UG3, UH2, UH3, UH5, UM1, UM2, U01, U19, and U34.^8,9^ Academic surgeon data by gender were retrieved from the Association of American Medical Colleges Physician Masterfile as previously described.^10,11^ Surgeon-scientists are academic surgeons who conduct clinical, translational, and/or basic science research in their career. While surgical research may be funded by many agencies, the NIH remains the largest source of biomedical and clinical research funding that simultaneous confers many career benefits.^3,4^ Therefore, for the purpose of this study, we defined surgeon-scientists as academic surgeons who received NIH funding and use the terms *surgeon-scientists* and *surgeon principal investigators* to refer to NIH-funded surgeons hereinafter.

### Demographic Variables

Information on each principal investigator was obtained via a web-based search of institutional and online networking service profiles, including academic degree obtained, board certification, gender identity, and year of first faculty appointment. A principal investigator was classified as a surgeon-scientist and therefore included in the study if they held an MD or both an MD and PhD and were board certified in one of the following surgical specialties: general surgery, colorectal surgery, neurological surgery, orthopedic surgery, ophthalmology, otolaryngology, plastic surgery, vascular surgery, cardiothoracic surgery, or urology. Surgeon-scientists’ gender identity was determined via personal pronouns used in institutional and online networking service profiles as well as institutional photographs. Year of first faculty appointment was obtained from institutional profiles and faculty curriculum vitae; if these sources were unavailable, the year was inferred as the year following fellowship completion.

### Outcomes

The Grant Support Index is an NIH-generated measure on a scale of 1 to 10 to evaluate funding support to principal investigators, with larger grant index indicating more monetary support.^12^ Large-dollar funding mechanisms are grants with a grant support index of 7 or higher, which includes DP1, DP2, DP3, DP4, P01, PN1, R01, R33, R35, R37, R56, RC4, RF1, RL1, RM1, UC1, UF1, UH3, UM1, UM2, and U01 grants.

Super principal investigators (SPIs) are investigators who are well funded by the NIH and therefore hold considerable career security and influence on policy making at both institutional and national levels.^5^ Principal investigators with competitive and high research funding are more advantageous in career advancement and promotion^13^ and are more likely to publish in prestigious journals.^14^ For the purpose of this study, an SPI was defined as a surgeon-scientist principal investigator with annual research funding equivalent to 2 R01 grants (ie, $750 000) or more as previously described.^15^

### Statistical Analysis

Statistical analysis was performed between April 1 and August 31, 2022. Funding dollars were inflation-adjusted to 2019 US dollars using the Biomedical Research and Development Price Index. Fold-change comparison was used to compare changes in funding and number of surgeon-scientists vs all academic surgeons by gender, and χ^2^ tests were used to determine significant differences in numbers between 1995 and 2020. The growth of grants and dollar funding to surgeon-scientists compared with all academic surgeons by gender were compared using simple linear regression, and differences in slopes were compared using *F* statistics. Time to first grant was compared across gender using *t* statistics. The χ^2^ test was used to determine differences in women and men surgeon-scientists with large-dollar grants or being a SPI. Because grant funding success varies by academic degree and research-intensiveness of institutions, adjusted relative risk of holding a large-dollar grant and being an SPI were computed using modified Poisson regression with robust error variance adjusting for academic degree (MD vs MD and PhD) and NIH research ranking of the principal investigator’s institution. All hypothesis tests were 2-sided with significance set at *P* < .05. Statistical analyses were performed in Stata, version 16.1 (StataCorp LLC).

## Results

**Figure 1.**
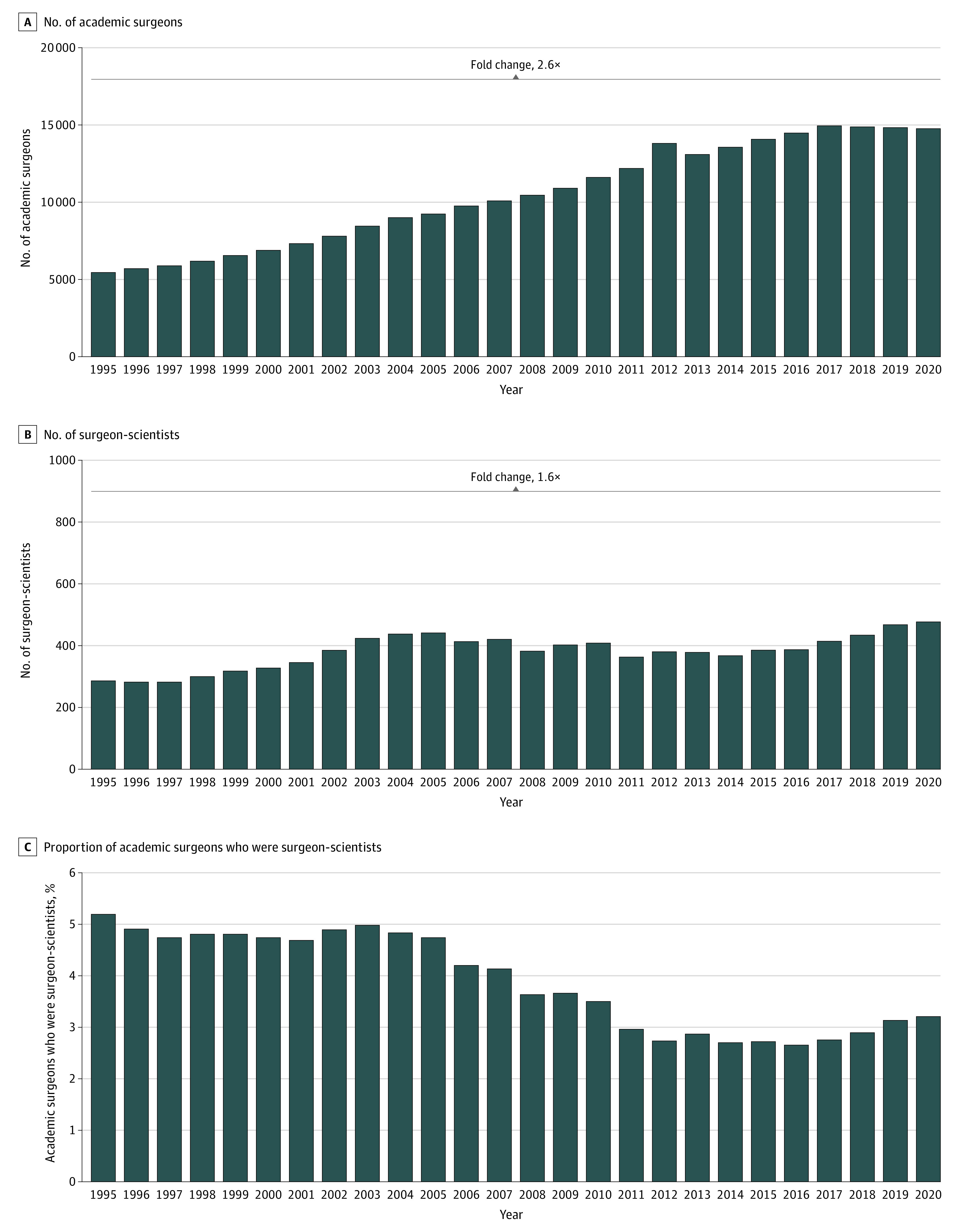
Distribution of Academic Surgeons and Surgeon-Scientists Who Were Principal Investigators (PIs), 1995 to 2020 A, Number of academic surgeons from 1995 to 2020. B, Number of surgeon-scientists from 1995 to 2020. C, Proportion of academic surgeons who were PIs from 1995 to 2020.

Between 1995 and 2020, a total of 2078 surgeons received research grant funding from the NIH. The number of academic surgeons increased 2.6-fold from 5611 to 14 927 (Figure 1A) compared with a 1.6-fold increase in surgeon-scientists, from 294 to 485 (Figure 1B) (*P* < .001). This resulted in a decrease in the relative proportion of surgeon-scientists among all academic surgeons from 5.2% to 3.2% (*P* < .001) (Figure 1C).

**Figure 2.**
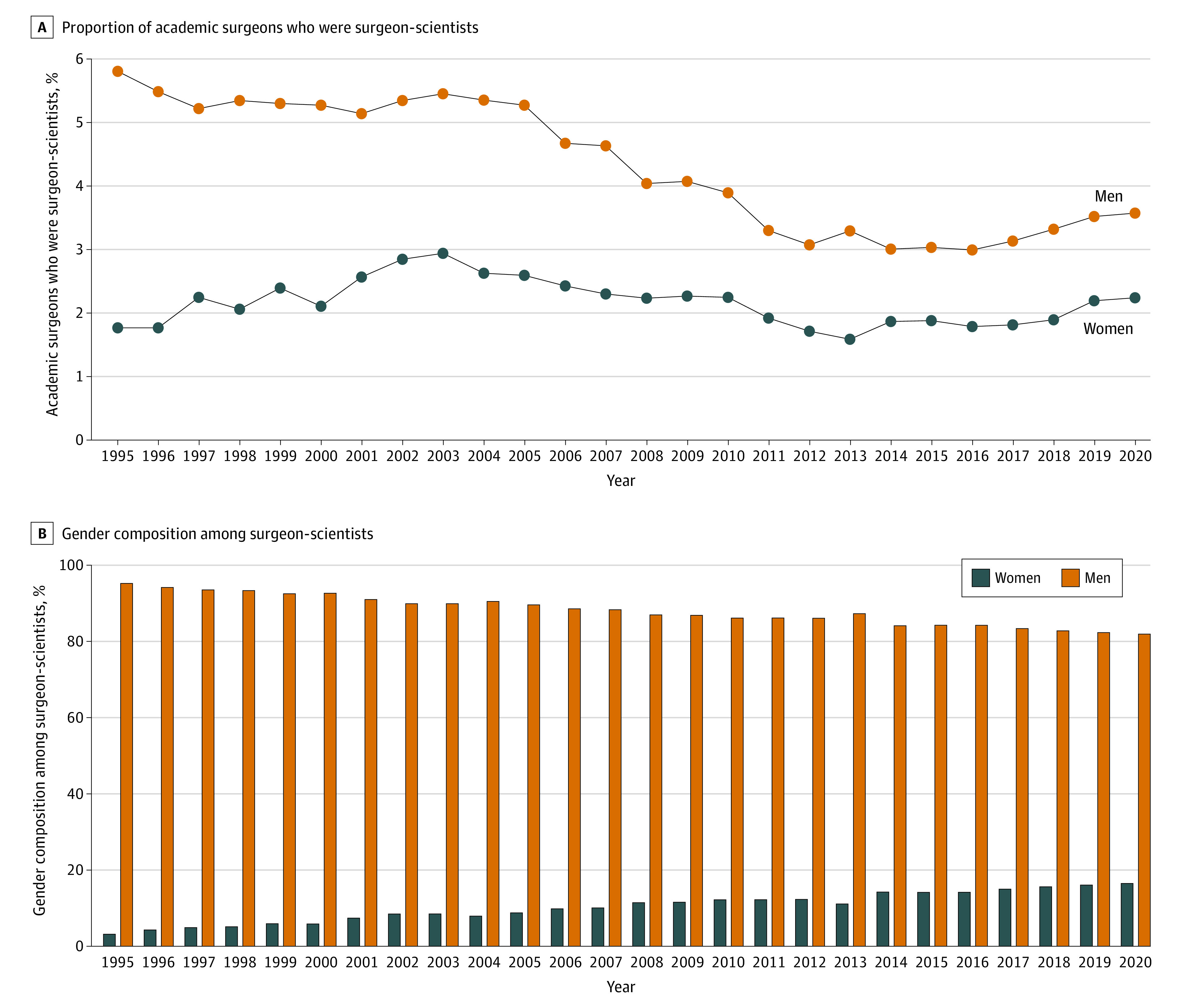
Gender Composition of Academic Surgeons Who Were Surgeon-Scientists, 1995 to 2020 A, Percentage of academic surgeons who were NIH-funded between 1995 and 2020 by gender. B, Distribution of surgeon-scientists between 1995 and 2020 by gender.

Between 1995 and 2020, the percentage of academic surgeons who were women increased significantly at a rate of 0.55% per year (14.1% in 1995 vs 27.9% in 2020; *P* < .001, data not shown). The percentage of surgeon-scientists who were women increased at a similar rate of 0.53% per year (4.7% in 1995 vs 18.8% in 2020; *P* < .001). The proportion of women academic surgeons who were surgeon-scientists increased significantly from 1995 to 2020 (1995, 14 of 792 [1.8%] vs 2020, 92 of 3834 [2.4%]; *P* = .10) (Figure 2A). The proportion of men academic surgeons who were surgeon-scientists decreased at a rate of 0.12% per year (from 280 of 4820 [5.8%] in 1995 to 393 of 10 989 [3.6%] in 2020; *P* < .001) (Figure 2A). Despite this decrease, of the 485 surgeon-scientists in 2020, only 92 (18.9%) were women (Figure 2B).

**Figure 3.**
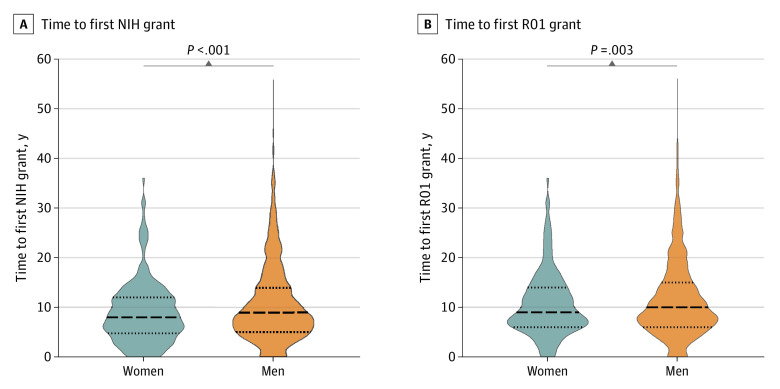
Mean Time to Receipt of First Research Grant From 1995 to 2020 by Gender A, Mean number of years to first National Institutes of Health (NIH) grant from year of first faculty appointment by gender. B, Mean number of years to first R01 NIH research project grant from year of first faculty appointment by gender. Dashed horizontal lines indicate the mean; dotted horizontal lines indicate the SD.

Time to first NIH grant was also significantly different by gender. The mean (SD) time to first research project grant of any mechanism for women surgeon-scientists was 2 years earlier than for men surgeon-scientists (8.8 [6.2] vs 10.8 [7.9] years, respectively; *P* < .001) (Figure 3A). Among R01 recipients, the mean (SD) time to receipt of first R01 grant after first faculty appointment was 1.1 years earlier for women surgeon-scientists compared with men surgeon-scientists (10.5 [6.4] vs 11.6 [7.8] years, respectively; *P* = .003) (Figure 3B).

**Figure 4.**
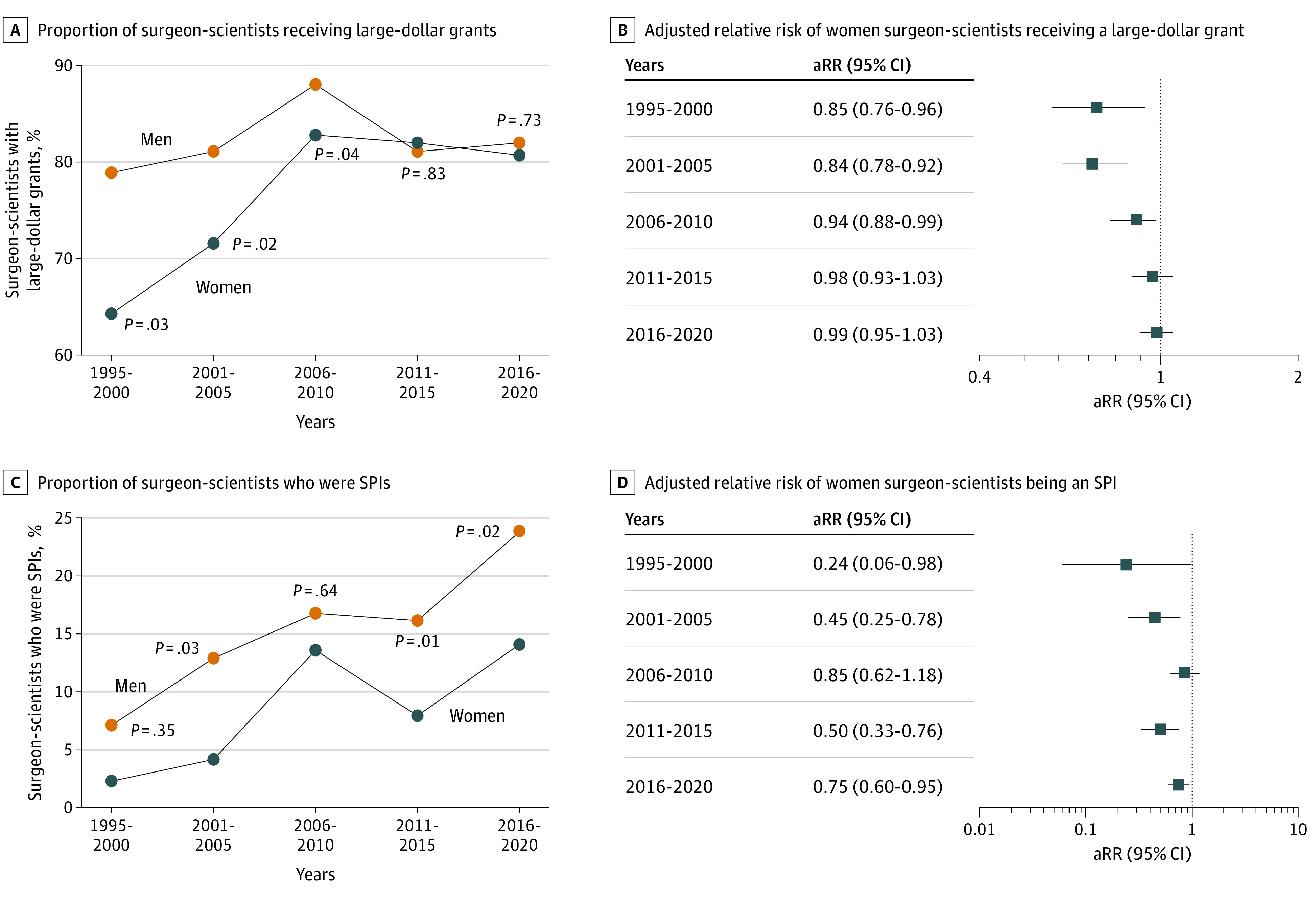
Distribution of National Institutes of Health Funding Among Surgeon-Scientists Who Were Principal Investigators by Gender A, Percentage of men and women surgeon-scientists with large-dollar grants in 5-year increments between 1995 and 2020. B, Adjusted relative risk of women surgeon-scientists having a large-dollar grant vs men surgeon-scientists, adjusting for academic degree (MD vs MD and PhD) and institutional National Institutes of Health (NIH) research ranking. C, Percentage of women and men surgeon-scientists who were super principal investigators (SPIs) with annual research funding totaling $750 000 or more in 5-year increments between 1995 and 2020. D, Adjusted relative risk of women surgeon-scientists being an SPI vs men surgeon-scientists, adjusting for academic degree (MD vs MD and PhD) and institutional NIH research ranking. aRR indicates adjusted relative risk.

Among surgeon-scientists, disparity in funding changed during the 25-year study period, with significant increase in the proportion of women surgeon-scientists receiving large-dollar grants. Between 1995 and 2000, 393 of 498 men surgeon-scientists (78.9%) received large-dollar grants while only 27 of 42 women surgeon-scientists (64.3%) received large-dollar grants (*P* = .02). Between 2016 and 2020, a similar proportion of women and men surgeon-scientists received large-dollar grants (109 of 135 [80.7%] vs 492 of 600 [82.0%], respectively; *P* = .73) (Figure 4A). After adjusting for academic degree and institutional NIH research ranking, women surgeon-scientists experienced improving odds of holding a large-dollar grant over time compared with men surgeon-scientists (1995-2000, aRR, 0.85 [95% CI, 0.76-0.96]; 2001-2005, aRR, 0.84 [95% CI, 0.78-0.92]; 2006-2010, aRR, 0.94 [95% CI, 0.88-0.99]) (Figure 4B). After 2010, women surgeon-scientists were as likely as men surgeon-scientists to obtain large-dollar grants (2011-2015, aRR, 0.98 [95% CI, 0.93-1.03]; 2016-2020, aRR, 0.99 [95% CI, 0.95-1.03]) (Figure 4B).

The percentage of surgeon-scientists who were an SPI increased during the 25-year study period (16 of 294 [5.4%] in 1995 to 126 of 485 [25.9%] in 2020; *P* < .001). Between 1995 and 2005, women surgeon-scientists were less likely to be an SPI compared with men surgeon-scientists (1995-2000, 1 of 42 [2.3%] vs 35 of 498 [7.0%], respectively; *P* = .35 and 2001-2005, 3 of 72 [4.2%] vs 78 of 605 [12.9%], respectively; *P* = .03) (Figure 4C). This gap decreased by the period 2006 to 2010 (12 of 88 [13.6%] vs 103 of 615 [16.8%], respectively; *P* = .64) (Figure 4C) but widened again between 2016 and 2020 (19 of 135 [14.1%] vs 143 of 600 [23.8%], respectively; *P* = .02) (Figure 4C). After adjusting for academic degree and institutional NIH research ranking, women surgeon-scientists remained significantly less likely to be an SPI compared with men surgeon-scientists by the period 2016 to 2020 (aRR, 0.75 [95% CI, 0.60-0.95]) (Figure 4D).

## Discussion

Although the representation of women among surgeon-scientists increased steadily during the 25-year period of this cross-sectional study, the relative proportion of women academic surgeons who were surgeon-scientists did not change. Despite a faster timeline to one’s first NIH grant, women surgeon-scientists remained significantly underrepresented among surgeon-scientists. This disparity was greatest among well-funded surgeon-scientists, where women surgeon-scientists were 25% less likely than men surgeon-scientists to be an SPI.

The surgeon-scientist workforce faces critical challenges due to increasing competitiveness of NIH funding, increasing clinical responsibility among surgeons, and lack of protected research time.^6,16,17,18,19,20^ Although these challenges affect both men and women surgeons, resulting in underfunding of surgeon-scientists,^16^ the persistent gender disparity in NIH funding suggests that these barriers may disproportionately affect women surgeons. A potential solution to the diminishing surgeon-scientist workforce is to invest in the career and development of women surgeon-scientists.^21^ In the present study, we found that women surgeon-scientists were as likely to hold large-dollar grants as men surgeon-scientists. Furthermore, women surgeon-scientists had a faster timeline to first grant funding compared with their men counterparts. This is a remarkable achievement that is not universal among the biomedical research workforce, where women and men biomedical scientists are of comparable age when obtaining their first R01 grant.^8^ Our findings are consistent with previously published findings that men and women surgeon-scientists have similar rates of converting NIH career development awards to R01 grants and similar numbers of R01 application attempts prior to success.^22^

Despite the success of women surgeon-scientists, the number of women surgeon-scientists is still relatively small compared with the proportion of women academic surgeons. A prior study concluded that there is an overrepresentation of women surgeon-scientists compared with academic surgeons.^23^ However, this study included only surgery departments, which may exclude other surgical specialties that are not as diverse as general surgery, such as orthopedic surgery and urology.^24^ Our findings suggest that the proportion of women academic surgeons who were funded by the NIH remained unchanged during the 25-year study period (1.8% vs 2.4%) and that the representation of women among surgeon-scientists remained low at 17.3%. In 2020, despite the rising interest of women medical trainees in surgery,^25^ women surgeon-scientists remained underrepresented among surgeon-scientists. If current trends persist, gender parity among surgeon-scientists will not be achieved until 2084. To facilitate this timeline, it is critical to address the barriers that women face in their pathway to a career as a surgeon-scientist.

One critical barrier is the lack of institutional support for women in surgery.^21^ Despite an increasing interest in surgery among women surgical trainees, women are still underrepresented among surgeon-scientists.^25^ Women surgeons often lack the critical mentorship necessary for advancement.^26,27^ Women surgery trainees are more likely to experience burnout and mistreatment compared with their men counterparts, resulting in higher attrition from surgical training.^28,29^ In addition, lack of support surrounding childcare and motherhood may also increase the risk of attrition from surgical training.^30^ Increasing support to facilitate the transition of women surgical trainees through their career path is key to retaining a gender-diverse surgeon-scientist workforce. Current excellent examples of programs to mentor women surgeons through their career transitions include the American College of Surgeons Women in Surgery Committee’s Mentorship Program and the Association of Women Surgeon’s Fellowship Grant for women surgeons. Furthermore, the Association of Women Surgeons recently published a guideline for a comprehensive support for women residents and fellows.^31^ These recommendations include adequate paid parental leave, insurance coverage, and access to childcare and should be incorporated into training policies by surgical specialty boards and surgical departments.

Another critical barrier for women surgeon-scientists is the pathway toward academic leadership. Women surgeons are significantly less likely to be in a leadership position than men surgeons, with only 30 women currently serving as a chair of a department of surgery.^11^ It is notable that this underrepresentation among surgical leadership is not due to lack of academic productivity, as we found that women surgeon-scientists were as successful as men surgeon-scientists in obtaining large-dollar grants but were significantly less likely to be an SPI. Thus, it is likely that despite their early research success, other barriers are present that impede the ascension of women surgeons to SPI status and other leadership roles. These barriers may include factors such as an exclusionary institutional culture that supports men surgeons as well as the traditional paradigm of work-life balance that does not support motherhood.^26^ Additionally, increasing women surgeon-scientists’ access to leadership positions is key to improving retention and recruitment of women surgeon-scientists.

While women surgeon-scientists maintained their success in NIH funding during the 25-year period analyzed in the present study, gender inequality in NIH funding persists.^8^ Although women scientists generate innovative research, their work is often devalued and receives fewer citations, leading to less recognition than men scientists.^1^ Similarly, in the field of surgery, despite publishing more papers, women surgeons are less likely to be cited than their men counterparts.^32^ Differential treatment and amplification of academic work by gender may also be reflected in NIH peer review.^33^ We found that women surgeon-scientists were 25% less likely than men surgeon-scientists to be an SPI, even after adjusting for academic degree and institutional NIH research ranking. Recent studies of US Department of Veteran Affairs study sections found that having more representation of women in study sections was associated with a higher likelihood of funding for applicants who are women.^34^ Increasing representation of women surgeon-scientists in study sections may improve equity and reduce bias in the NIH funding process.

### Limitations

Our study has several limitations. Our analysis lacked data on grant application behavior among surgeon-scientists. Grant application success rate is a factor driving the gender distribution among surgeon-scientists, and reduced numbers of funded grants may reflect multiple factors preventing women from submitting similar numbers of grant proposals as men. In addition, surgeons often receive funding from federal sources other than the NIH, such as the US Department of Veteran Affairs and US Department of Defense. Additionally, although we did not have access to race and ethnicity data, previous studies have shown that racial and ethnic minority women face additional challenges in pursuing surgery and research.^35^ Consideration of intersectional identity in future studies is key to generating a gender-diverse surgeon-scientist workforce.

## Conclusions

This cross-sectional study found that the representation of women surgeon-scientists remained low during the period 1995 to 2020. While surgeon-scientists face critical challenges in the current hypercompetitive NIH funding climate, the capacity of women surgeon-scientists to obtain NIH funding early in their career is promising. Increasing gender diversity among surgeon-scientists may prove to be critical in promoting the surgeon-scientist workforce and improving diversity within the surgery research enterprise.

## Notes

Supplement.
Data Sharing Statement
