# Supplementary material for: Gender Disparity in National Institutes of Health Funding Among Surgeon-Scientists From 1995 to 2020
Source: JAMA Netw Open. 2023 Mar 20;6(3):e233630. doi: 10.1001/jamanetworkopen.2023.3630 (PMC10028489; doi:10.1001/jamanetworkopen.2023.3630)
Supplement: Supplement. — Data Sharing Statement [file jamanetwopen-e233630-s001.pdf]

Data Sharing Statement

Nguyen. Gender Disparity in National Institutes of Health Funding Among Surgeon-Scientists From 1995 to 2020. *JAMA Netw Open*. Published March 20, 2023.
doi:10.1001/jamanetworkopen.2023.3630

Data
Data available: No
